# The Lion in West Africa Is Critically Endangered

**DOI:** 10.1371/journal.pone.0083500

**Published:** 2014-01-08

**Authors:** Philipp Henschel, Lauren Coad, Cole Burton, Beatrice Chataigner, Andrew Dunn, David MacDonald, Yohanna Saidu, Luke T. B. Hunter

**Affiliations:** 1 Panthera, New York, New York, United States of America; 2 Environmental Change Institute, University of Oxford, Oxford, United Kingdom; 3 School of Geography and Environmental Planning, University of Queensland, Brisbane, Australia; 4 Alberta Biodiversity Monitoring Institute, University of Alberta, Edmonton, Canada; 5 Biology Department, University of Victoria, Victoria, British Columbia, Canada; 6 IUCN, Protected Area Program, Ouagadougou, Burkina Faso; 7 Wildlife Conservation Society, Nigeria Program, Calabar, Nigeria; 8 Wildlife Conservation Research Unit, Department of Zoology, University of Oxford, The Recanati-Kaplan Centre, Oxford, United Kingdom; 9 Nigeria National Park Service, Garki-Abuja, Nigeria; Bangor University, United Kingdom

## Abstract

The African lion has declined to <35,000 individuals occupying 25% of its historic range. The situation is most critical for the geographically isolated populations in West Africa, where the species is considered regionally endangered. Elevating their conservation significance, recent molecular studies establish the genetic distinctiveness of West and Central African lions from other extant African populations. Interventions to save West African lions are urgently required. However formulating effective conservation strategies has been hampered by a lack of data on the species' current distribution, status, and potential management deficiencies of protected areas (PAs) harboring lions. Our study synthesized available expert opinion and field data to close this knowledge gap, and formulate recommendations for the conservation of West African lions. We undertook lion surveys in 13 large (>500 km^2^) PAs and compiled evidence of lion presence/absence for a further eight PAs. All PAs were situated within Lion Conservation Units, geographical units designated as priority lion areas by wildlife experts at a regional lion conservation workshop in 2005. Lions were confirmed in only 4 PAs, and our results suggest that only 406 (273–605) lions remain in West Africa, representing <250 mature individuals. Confirmed lion range is estimated at 49,000 km^2^, or 1.1% of historical range in West Africa. PAs retaining lions were larger than PAs without lions and had significantly higher management budgets. We encourage revision of lion taxonomy, to recognize the genetic distinctiveness of West African lions and highlight their potentially unique conservation value. Further, we call for listing of the lion as critically endangered in West Africa, under criterion C2a(ii) for populations with <250 mature individuals. Finally, considering the relative poverty of lion range states in West Africa, we call for urgent mobilization of investment from the international community to assist range states to increase management effectiveness of PAs retaining lions.

## Introduction

The lion (*Panthera leo*) was the most successful large carnivore during the late Pleistocene, when the species' range extended from South Africa, across Eurasia, and into the southern United States [1]. Today, the lion's range is restricted to Africa and one population of the Asiatic sub-species, *P. l. persica*, in India [2]. While the endangered Asiatic population is currently considered stable, lion populations in Africa are in decline and the African sub-species, *P. l. leo*, is considered vulnerable [3]. Recent analyses established that the African lion has lost at least 75% of its original habitat, with fewer than 35,000 wild African lions remaining [4]. The main drivers of lion declines are large-scale habitat conversion, prey base depletion through unsustainable hunting, and the retaliatory killing of lions due to perceived or real human-lion conflict [3]. The situation is most critical in West Africa, where lions have been considered regionally endangered since 2004 [5], and where <500 individuals may persist [4].

West African lions represent a population with unique genetic and conservation value. Recent molecular and morphological data covering the species' entire historical range suggests that lions in Central, West and North Africa (the latter now extinct) are distinct from lions in Eastern and Southern Africa and share a common ancestor with lions in Asia [6]–[8]. These results establish that the principal subdivision of modern lions is within Africa [9], and question the current dichotomous division into an African and an Asian sub-species; a division which is still widely supported, including by the IUCN Red List [3]. Moreover, they demonstrate that lions in West Africa contain mtDNA haplotypes not found in other lion populations, elevating the conservation significance of the few remaining West African populations [8], [9].

Conservation interventions to save these populations are now urgently required. However, formulating interventions is limited by few field data on the species' current distribution, abundance, and predominant drivers of declines in West Africa. While lions have been the object of extensive research effort in parts of Eastern and Southern Africa, they have been largely ignored in West Africa. Of 463 articles on African lions in the ISI Web of Science™ (Thompson Scientific) in 2005, not one focused specifically on lions in West Africa. To address this deficit, we first collated available data and expert opinions on lion distribution and status in West Africa. We then undertook field surveys in 13 large (>500 km^2^) protected areas (PAs) where lions were reported during this process, to determine lion presence/absence and estimate lion population size. For a further eight large PAs suspected to harbor lions, we compiled field survey data from the literature and via interviews. Using standardized evaluation toolkits for protected area management effectiveness [10], we compared current management performance of PAs known or suspected to harbor lions and those from which lions have likely been extirpated. With these data, we provide a comprehensive evaluation of the tenuous status of the West African lion, and make recommendations for the taxa's conservation.

### Study area

**Figure 1:**
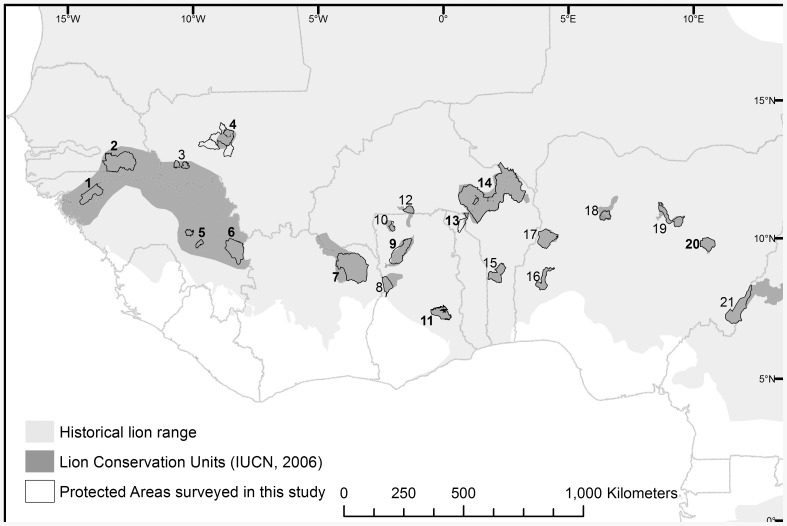
Lion Conservation Units [18] and surveyed protected areas (PAs) in West Africa. Number symbols in the map correspond to PA identification numbers in Table 1 and Tables S3/S4, with numbers printed in bold representing PAs with METT assessments.

We restrict our analysis to West Africa, as defined by the United Nations geoscheme (http://unstats.un.org/unsd/methods/m49/m49regin.htm), including all countries from Senegal to Nigeria (Fig. 1). The same classification was used for the listing of the lion as regionally endangered in West Africa [5]. Historically, lions occurred in all biomes in West Africa, with the exception of the coastal Upper and Lower Guinean Forests and the interior of the Saharan Desert (Fig. 1). The collapse of lion range in West Africa is poorly documented, but appears to be linked to large-scale habitat loss outside PAs through conversion to agriculture [5]. Consequently, lion range in this region is largely restricted to PAs [4], [11]. While several West African countries have large PAs, average PA performance in West Africa ranked poorest in a cross-continental comparison: large mammal populations in eleven West African PAs declined by an average of 85% between 1970–2005, compared to an average 59% decline across the continent [12]. Population collapse within these PAs appears to be driven by commercial bushmeat exploitation, supplying local markets in West Africa [13]–[15], and has contributed to bringing several iconic large mammal species to the brink of extinction in their West African range [16], [17].

## Methods

### Consultation of expert opinion on current lion range

In 2005, the IUCN and the Wildlife Conservation Society (WCS) organized a lion conservation workshop for wildlife authorities from all lion range countries within West and Central Africa [18]. The workshop consisted of a technical session to map current lion range and status, followed by a strategic planning session to develop lion conservation strategies [18]. The technical session was modeled after the Range Wide Priority Setting process developed by WCS for jaguars [19]. Experts were guided to produce maps of current lion range and delimit areas harboring known or suspected populations called Lion Conservation Units (LCUs) [18]. LCU delimitation relied on credible evidence of lion presence within the preceding 10 years [18], and for each LCU participants assigned lion population trends and approximate lion population size. Although the data presented at the 2005 workshop now date from 1995 onwards, at the onset of our field surveys in 2006 (see below), it represented the most comprehensive and reliable dataset on lion distribution in West Africa.

### Field surveys

Between October 2006 and May 2012, we conducted field surveys in PAs within designated LCUs, to 1) confirm lion presence for PAs where evidence of lion occurrence was lacking, and 2) establish lion population estimates for PAs where lions occurred. Although designated LCUs included both protected and non-protected areas, lions are largely absent outside PAs in West Africa [11], [20]. Accordingly, we restricted fieldwork to PAs (IUCN PA categories I–VI) within LCUs (henceforth: LCU PAs), including two PAs awaiting formal designation (Table 1). A primary determinant of lion extinction risk within a given PA is its size, and we therefore restricted our survey effort to large (>500 km^2^) LCU PAs, based on findings on critical PA size for lions from prior studies [21], [22]. In LCUs with multiple PAs, we concentrated survey efforts in the largest PAs with the highest protection status (according to IUCN PA categories).

**Table 1:** Summary of recent survey activities in LCU PAs, permitting the assessment of lion population status.

Map ID	Name of protected area	Country	Area [km^2^] (IUCN PA category)	Year surveyed	Target species	Type of survey	Conclusion regarding lion occurrence	Lion pop size	Source
1	Boé and Dulombi NPs	Guinea-Bissau	1,943 (awaiting gazetting)	2010-ongoing	chimpanzee	camera trap and foot surveys	considered absent	n.a.	J. van Schijndel (www.chimbo.org), pers. comm.
2	Niokolo-Koba NP	Senegal	9,130 (II)	2011	lion	track surveys (by vehicle)	confirmed present	16 (0–54)	this study
3	Bafing-Faleme	Mali	3,326 (1094 II+672 IV+1561 none)	2003–2004	chimpanzee	foot surveys	considered absent	n.a.	Granier & Martinez [54]
4	Boucle de Baoulé NP	Mali	5,330 (II)	2012	faunal inventory	foot surveys	considered absent	n.a.	B. Niagate, pers. comm.
5	Haut-Niger NP	Guinea	1,228 (II)	2009	lion	call-up and track surveys (on foot)	potentially present	n.a.	PFNH [55]
6	Kankan Faunal Reserve	Guinea	5,314 (IV)	2007	faunal inventory	foot surveys	potentially present	n.a.	Dufour [47]
7	Comoé NP	Cote d'Ivoire	11,495 (II)	2010	lion	track surveys (on foot)	considered absent	n.a.	this study
8	Bui NP	Ghana	1,897 (II)	2009	lion	interview survey	considered absent	n.a.	this study
9	Mole NP	Ghana	4,522 (II)	2006–2009	lion	camera trap and track surveys (by vehicle and on foot)	considered absent	n.a.	this study
10	Gbele Resource Reserve	Ghana	544 (IV)	2008	lion	camera trap and track surveys (on foot)	considered absent	n.a.	this study
11	Digya National Park	Ghana	2,789 (II)	2009	lion	interview survey	considered absent	n.a.	this study
12	Nazinga Game Ranch	Burkina Faso	970 (none)	1985-ongoing	faunal inventory	foot surveys	considered absent	n.a.	Delvingt & Vermeulen [56]
13	Oti-Mandouri National Park	Togo	1,100 (II)	2003	faunal inventory	aerial surveys^a^	considered absent	n.a.	Bouché et al. [57]
14	W-Arly-Pendjari	Benin/Burkina Faso/Niger	27,167 (14,629 II+10,728 IV+1,809 VI)	2012	lion	track surveys (by vehicle)	confirmed present	356 (246–466)	this study
15	Mt Kouffe/Wari Maro	Benin	3,092 (VI)	2012	lion	consolidation of expert opinion	considered absent	n.a.	CENAGREF [58]
16	Old Oyo National Park	Nigeria	2,386 (II)	2009	lion	interview survey	considered absent	n.a.	this study
17	Kainji Lake National Park	Nigeria	3,970 (II)	2011	lion	call-up surveys	confirmed present	32 (23–63)	this study
18	Kamuku National Park	Nigeria	1,121 (II)	2009	lion	interview survey	considered absent	n.a.	this study
19	Falgore and Lame-Burra Game Reserves	Nigeria	2,910 (IV)	2009	lion	track surveys (on foot)	considered absent	n.a.	this study
20	Yankari Game Reserve	Nigeria	2,244 (IV)	2011	lion	call-up surveys and ranger-based lion monitoring	confirmed present	2	this study
21	Gashaka-Gumti National Park	Nigeria	1,900 (II)^b^	2009	lion	track surveys (on foot)	considered absent	n.a.	this study
Total			99,148 (94,378 suitable for lion)					406 (250–587)	

For more details on the surveys, see Table S3.

^a^ Aerial surveys established complete absence of large wild herbivores, strongly indicating PAs unsuitability for lions.

^b^ Gashaka-Gumti NP encompasses 6,670 km^2^, however, only ca 1,900 km^2^ comprise suitable lion habitat.

We also compiled data on lion presence from recent field surveys led by other institutions, from internal reports and interviews with participants. We included only surveys that targeted large mammals, with survey methods and effort appropriate to detect lions. Finally, we incorporated data from interviews of PA staff on lion presence/absence for several LCU PAs that have not been recently surveyed. We did not consider reports of lion presence without physical evidence and records >10 years old.

### Establishing lion presence/absence

Survey methods commonly used for African savannah mammals, such as aerial surveys or line transects, typically yield few observations of large terrestrial carnivores [23]. Consequently, prior efforts to establish large carnivore occurrence and/or abundance over large spatial scales relied on interviews [24], remote cameras [25], or track surveys [26]. We predominantly employed track surveys, owing to their comparatively high detection efficiency, and low effort and cost [26]. All surveys teams included experienced observers, and we ascribed field sign to species based on pugmark characteristics [27]. Occasionally teams found equivocal tracks, mainly regionally rare species such as African wild dog (*Lycaon pictus*) and cheetah (*Acinonyx jubatus*). In such cases we documented pugmarks photographically, and presented photos to a panel of experts for species verification. We omitted a small number of records from analysis if they could not be assigned to species unequivocally. In PAs with an intact network of dirt tracks, we conducted vehicle-based track surveys, with two observers seated on the bull-bar of a vehicle driven at a maximum speed of 10–20 km h^−1^ [28]. In PAs without penetrable roads, we conducted track searches on foot along roughly predefined survey circuits, following game trails, dry riverbeds, abandoned dirt tracks or other linear features commonly used as travel routes by lions and other large carnivores. These circuits incorporated habitat features that could be expected to attract larger herbivores, such as water reservoirs, floodplains, saltlicks and marshes, or other sites with high herbivore abundance indicated by PA staff. In Mole NP and Gbele Resource Reserve in Ghana, camera traps were the primary survey method. At those PAs, we deployed DeerCam DC-300 (Non Typical, Wisconsin, USA) camera units at ∼1 km intervals, targeting, as with foot surveys, features expected to maximize lion capture probability [29]. In Mole NP, we concentrated trapping effort in the central and southeastern portions known to contain higher prey densities and key dry-season water sources. In Comoé National Park (NP) in Côte d'Ivoire, West Africa's largest NP at >10,000 km^2^, we conducted an aerial survey prior to our ground survey, to identify areas with important concentrations of potential lion prey. We restricted the ensuing foot survey to those areas.

### Estimating lion population size

If lions were found in a given PA, we used systematic track counts [28] or call-ups [30] to estimate lion population size, depending on local conditions. Neither method has ever been locally calibrated in West Africa. However, we preferred track counts due to the generally consistent relationship between lion track densities and actual lion densities observed across a wide range of different climatic and habitat-related conditions in Eastern and Southern Africa [28]. One important caveat of this method is that the relationship between track densities and actual densities varies with substrate type, and we therefore recorded substrate type every 500 meters along spoor transects, to enable us to select the appropriate relationship from Funston et al [28]. In two PAs with lion presence where the poor state of the road network precluded the use of vehicle-based track counts, we conducted call-ups to estimate lion population size. The call-up method requires calibration experiments to assess local response distance and response rate of lions to broadcasts [30]. Because lion observations were extremely rare during our surveys, we obtained only one response distance estimate in dense Sudano-Sahelian woodland; one male lion encountered opportunistically 2.5 km from a call-up station, was observed again at the station 26 minutes after the onset of the broadcast, fifty minutes after the initial observation. As the male was traveling in the opposite direction when first seen, we consider this a conservative estimate for a maximum response distance in dense woodland, and used this value here. To derive tentative estimates of lion population density based on our call-up results, we used the range of published figures on lion response rates [30], [31]. Study design and data analysis of our systematic track counts followed Funston et al. [28], while our protocol for call-ups followed Ferreira and Funston [31]. All field surveys were carried out in close collaboration with the respective national wildlife authorities, and involved senior PA research staff. Our survey work was therefore considered an integral part of preexisting PA monitoring activities, and wildlife authorities waived requirements for formal research clearance and PA entry fees. All field methods used were completely noninvasive and did not require the handling or sampling of live animals, and our survey work did therefore not require approval from an ethics committee.

### Evaluating management of LCU PAs

Prior studies highlighted the strong impact of PA characteristics (e.g. PA size), human population density at PA edges, and PA management variables (e.g. PA management budgets) on lion persistence and population status inside PAs [21], [32], [33]. Therefore, we investigated correlations between lion persistence and a number of continuous PA variables, including annual budget, staff number, area, surrounding human population density and IUCN management category, using univariate analyses (Analysis of Variance (ANOVA) and Mann Whitney U tests) where sample size allowed. We tested for univariate correlations between the PA variables using univariate linear regression or spearman's rank. We estimated human population density within a 5 km buffer around each PA based on human population data from the AfriPop Project (www.afripop.org), using PA outlines obtained from the World Database of Protected Areas (http://protectedplanet.net/). We extracted continuous management variables from WWF/World Bank Management Effectiveness Tracking Tool (METT) assessments carried out in the region. The METT is one of the most widely used assessment tools for Protected Area Management Effectiveness (PAME) [34], and is designed to be completed by PA managers, staff and stakeholders. Besides provisioning of continuous PA management variables such as budgets and staff numbers, the methodology encompasses a rapid assessment based on a scorecard questionnaire of 30 questions, with an ordinal four-point scale (0–3, with 3 representing the best management scenario). The complete METT questionnaire template is provided in Supporting Information S1, and Table S1 summarizes the 13 METT scored questions included in our analysis; we selected management aspects likely to influence the ability of a PA to enforce regulations and reduce hunting pressure, to provide insight into the managers' perception of current PA performance). A comprehensive list of all PA management variables used in our analyses, variable provenance, and corresponding sample sizes can be found in Table S2. All statistical analyses were performed in R [35].

## Results

### Lion presence and absence in West African PAs

**Figure 2:**
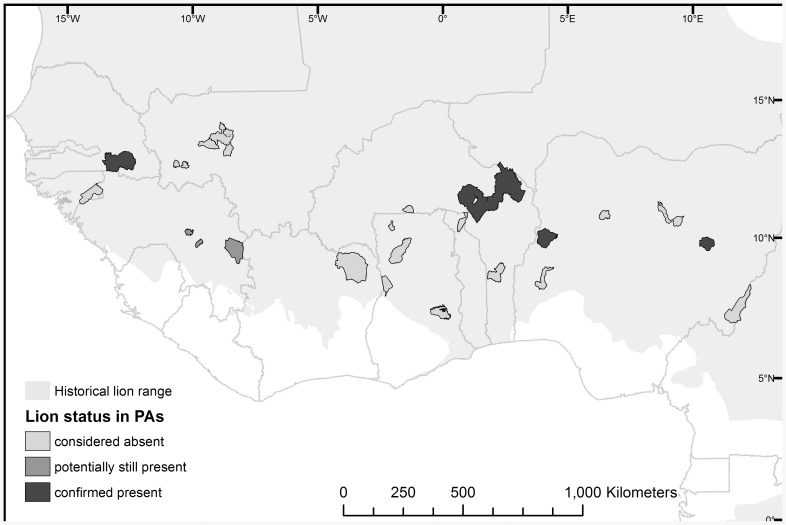
Lion status in West African protected areas within lion range.

Wildlife experts attending the 2005 workshop identified 17 LCUs in West Africa (Fig. 1), totaling 254,430 km^2^, or 5.8% of historic lion range in West Africa. We identified 21 large (>500 km^2^) PAs within those LCUs (Fig. 1, Table 1), with a total area of ∼95,000 km^2^, or 37% of total LCU extent. We surveyed thirteen of those PAs for lions, while the remaining PAs were surveyed by other researchers focusing on lions (n = 2), Western chimpanzees *Pan troglodytes ssp. verus* (n = 2), and general faunal inventories (n = 4) (Table 1). Of the 21 LCU PAs surveyed, lions were confirmed in only four (Table 1; Fig. 2). In two additional PAs, both in Guinea, lions had not been observed for >10 years; however, credible reports of vocalizations suggest they may still be present. Among the four PAs in which lion persistence was confirmed, three contain <50 individuals, and the only large population is in the W-Arly-Pendjari (WAP), with an estimated 356 (range: 246–466) lions (Table 1). The total number of lions remaining in West Africa is estimated at 406 (range: 250–587) individuals, while the confirmed lion range (the total size of PAs where lions were confirmed, including potential sites in Guinea) is estimated at 49,000 km^2^, or 1.1% of historic lion range in West Africa.

### Management of LCU Protected Areas

We identified METT assessments for 12 of the 21 LCU PAs (Table S2). Details of individual assessments can be found in Table S4. Due to the small sample sizes, we have not attempted multivariate analyses, and have presented statistical correlations only where continuous data (on PA budgets, staffing, area and human population density), as opposed to ordinal scores, were available.

**Figure 3:**
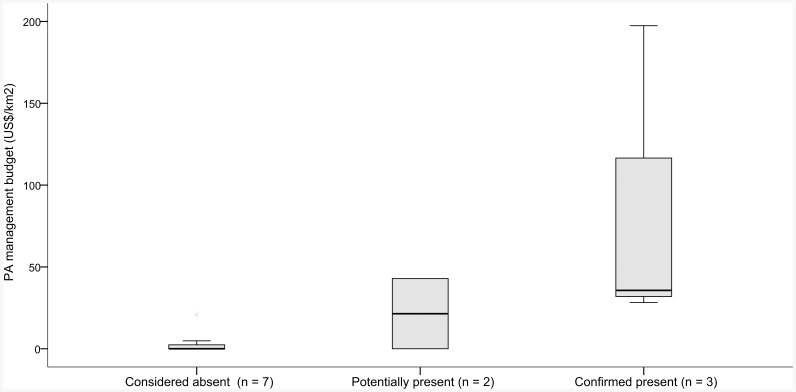
Boxplot illustrating median operating budgets (US$/km^2^) for PAs grouped by current lion population status. Hinges represent the 1st and 3rd quartiles, and whiskers represent the range of the data. Black points represent outliers.

Protected area budget (measured as total budget (US$) and budget/area (US$/km^2^)) was positively associated with lion persistence (Fig. 3), and PAs with lions were, on average, more than twice as large as those without, although the latter difference was not statistically significant, possibly due to the small sample (Table 2). Total PA budget significantly increased with area (spearman's rank coefficient = 0.65 p = 0.02).

**Table 2:** PA management characteristics by lion presence and absence (continuous data).

Management characteristics	All PAs	PAs where lions confirmed or potentially present	PAs where lions absent	Statistical test: Mann Whitney U (MW); ANOVA (AN).
	Mean (SE) (n)	Mean (SE) (n)	Mean (SE) (n)	
PA area (km^2^)	4,721 (1,275) (21)	8,175 (3,962) (6)	3,340 (730) (15)	Non-sig (AN); p = 0.13
Human population density (people within a 5 km buffer)	0.23 (0.05) (21)	0.21 (0.09) (6)	0.24 (0.06) (15)	Non-sig (AN); p = 0.92
PA staff/100 km^2^	1.45 (0.46) (12)	1.63 (0.62) (5)	1.32 (0.69) (8)	Non-sig (AN): p = 0.72
PA patrol staff/100 km^2^	0.86 (0.30) (12)	0.81 (0.26) (5)	0.91 (0.53) (8)	Non-sig (AN): p = 0.76
	Median (IQR) (n)	Median (IQR) (n)	Median (IQR) (n)	
PA budget (total US$)	6,746 (0–117,076) (12)	185,000 (80,000–257,742) (5)	0 (0–6746) (8)	Sig (MW): 29.5, p = 0.048
PA budget/area (US$/km^2^)	2.42 (0–30.1) (12)	35.7 (28.2–42.9) (5)	0 (0–2.41)(8)	Sig (MW): W = 30.5, p = 0.03
	Percentage	Percentage	Percentage	
PA category (II/IV/VI/none)	66.3/23.7/5.2/4.7 (21)	59.0/37.3/3.7/0.0 (6)	74.2/9.1/6.8/9.9 (15)	n/a

**Figure 4:**
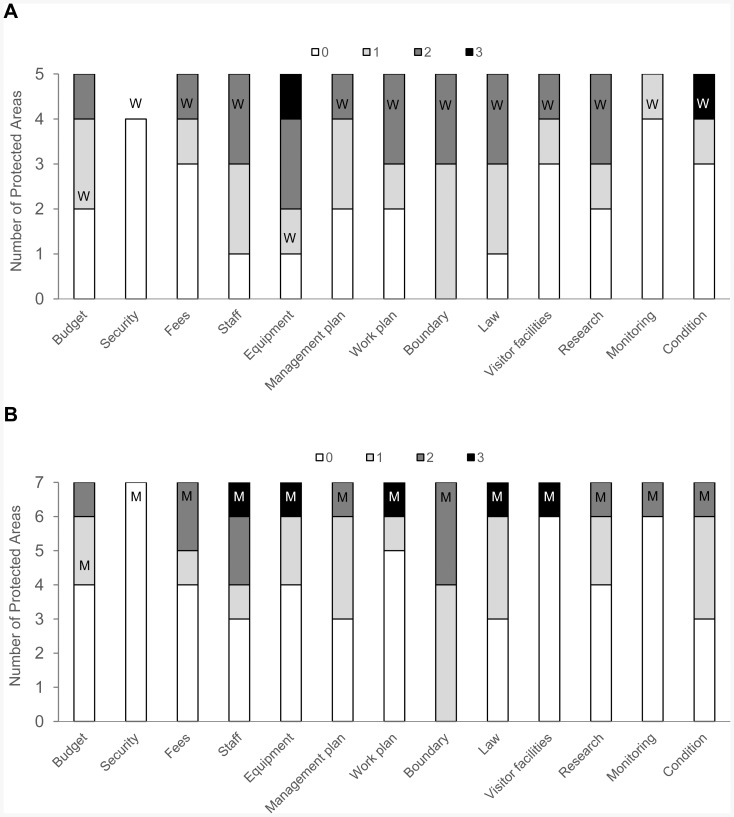
METT (Management Effectiveness Tracking Tool) scores for evaluated protected areas (PAs) in West Africa. (A) PAs where lions are confirmed present or are potentially still present (A); (B) PAs where lions were considered absent. The letter W represents scores for W-Arly-Pendjari while M indicates those for Mole NP (see text). Management scores range from 0–3, with 3 representing the best management scenario. For example, in the case of ‘Current Budget’ 0 = No PA budget; 1 = inadequate budget which creates serious management constraints; 2 = acceptable budget, but could be further improved to fully achieve effective management; 3 = sufficient budget which fully meets the needs of the PA. See Table S1 for full descriptions of scores.

METT scores indicate that the majority of PAs are experiencing severe management deficiencies over most facets of PA management (Fig. 4). Scores for only two PAs, WAP (where lions are present) and Mole (where lions are absent), suggest that they were being managed adequately (Fig. 4).

## Discussion

**Figure 5:**
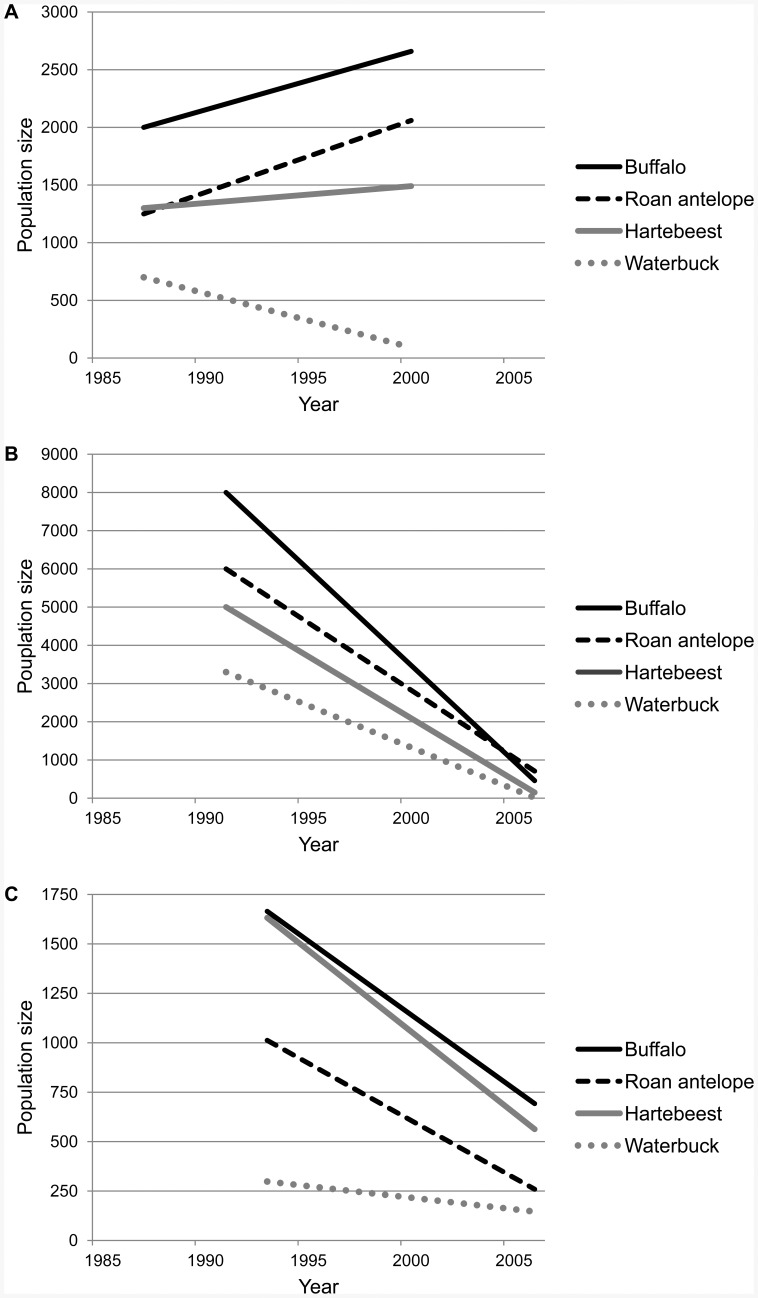
Populations trends for principal lion prey species in West African protected areas. (A) Pendjari NP (which forms part of W-Arly-Pendjari); (B) Niokolo-Koba NP; (C) Mole NP. Data sources: Galat et al. [59], Sinsin et al. [60], Wildlife Division of Ghana [61], Bouché [62], Renaud et al. [63].

The lion has undergone a catastrophic collapse in West Africa. Our results suggest that lions have lost almost 99% of their historic range, and that only ca. 400 individual lions persist across the region. Most of these lions (ca. 350 individuals, or 88% of the total population) persist in a single population in WAP, and there is strong evidence for ongoing declines in the region's other three populations. In Nigeria, numbers dropped from an estimated 44 lions in 2009 to 34 in 2011 [36]. In Senegal's Niokolo-Koba NP, continuing calamitous declines in prey populations (Fig. 5B) are almost certainly causing concomitant declines in lions. These trends suggest that WAP already or will soon contain >90% of West African lions. Given that 40–60% of a lion population typically consists of immature individuals [37], [38], and that our track counts in WAP included large cubs and sub-adults, it is very likely there are <250 adults remaining in the entire West African region. Accordingly, our results warrant listing of the lion as critically endangered in West Africa under criterion C2a(ii), which applies for declining populations with <250 mature individuals, where >90% of individuals persist in one subpopulation [39].

### Priorities for lion conservation in West Africa

Our surveys covered all large (>500 km^2^), formally designated PAs within LCUs in West Africa. While lion range in this region is largely restricted to PAs [4], [11], we cannot exclude the possibility that some lions roam outside the surveyed PAs. However, the 21 LCU PAs covered in this study represent the best remaining lion habitat in West Africa [4]. We deem survey effort adequate (see Table S3) to draw inference on the occurrence of resident lions across sites, and we are confident that no resident lion populations were overlooked by our efforts. Further survey work may be required in Haut Niger NP and Kankan FR in Guinea to assess the possible presence of lions. However, given the lack of physical evidence for over a decade and the poor management scores of those two PAs (see Table S4 and below), we believe any remaining populations would be relict and close to extinction. While continued survey and monitoring work is warranted, the highest conservation priority for lions in the region should be strengthening protection of the known remaining populations.

Lions are more likely to disappear from small PAs than from larger ones. Critical PA size for lions based on data from East Africa is 291 km^2^, using an average lion density of 16.2 adults 100 km^−2^ [22]. In comparison, average density across our four sites with lions was 1.0 lions 100 km^−2^, >15 times lower than in East Africa. Assuming the same lower limit for a viable lion population size in West Africa, critical PA size would exceed 4,000 km^2^ at current lion densities and even that may be inadequate. Newmark [40] revealed that extinction rates in Ghanaian PAs were estimated to be 13–77 times higher than in equivalent-sized PAs in Tanzania, suggesting that larger size alone a may provide insufficient protection against the intense hunting pressure impacting West African PAs. In addition to the lower carrying capacity of West African savannas for large herbivores [41], higher extinction risks for West African mammals is driven by intense bushmeat hunting pressure within and adjacent to PAs [13], [14], facilitated by ineffective PA management (Fig. 4) [12]. Our findings highlight the urgent need for very large (>4,000 km^2^) and well protected PAs to assure the survival of lions and other threatened large mammals in West Africa. Three of four extant lion populations in West Africa occur in PAs close to or larger than 4,000 km^2^ (Table 1), representing the best prospects for saving the taxon.

WAP currently harbors the only population >50 animals, and is the most viable. However, lion population density is extremely low in the eastern half of WAP, i.e. the tri-national W NP (Henschel et al. in prep). An aerial survey covering W immediately following our lion survey in 2012, recorded >50,000 head of cattle inside the national park, underlining the weak management effectiveness in W NP [42]. In contrast, the western half, Arly-Pendjari, supports higher lion densities, stable or increasing prey populations (Fig. 5A), and incursions by livestock into the PA are rare [42].The stark contrast in management effectiveness between the eastern and western halves of WAP may be due to the disparity in management budget allocations; of the US$197/km^2^ available for the WAP in aggregate, 90/km^2^ are spent in W, compared to 323/km^2^ in Arly-Pendjari (Henschel et al. in prep). A significantly higher operational budget is required in W to attain conservation outcomes comparable to Arly-Pendjari.

Lion populations in Niokolo-Koba and Kainji Lake NPs are small and appear to be declining. While no data on management effectiveness and lion prey populations exist for Kainji Lake NP, management effectiveness scores are low in Niokolo-Koba NP, potentially due to inadequate funding (Table S4), and prey populations have collapsed to extremely low levels over the past 20 years (Fig. 5B). Both PAs hold great potential due to their large size, and are surrounded by suitable lion habitat and moderate human population densities (Table S3) [4]. Furthermore, Kainji Lake NP is potentially still connected to WAP, through suitable lion habitat in Benin [4]. Besides lions, Niokolo-Koba also harbors the last important population of the critically endangered Western giant eland (*Tragelaphus derbianus ssp. derbianus*) [43], and the only confirmed population of the critically endangered West African sub-population of African wild dogs (Table S3) [44]. Both PAs will require immediate financial and technical assistance to avert the local extirpation of lions and other critically endangered taxa.

At 2,244 km^2^ Nigeria's Yankari Game Reserve is smaller than our putative minimum and its lion population is very small and declining (Table 1). Yankari is completely surrounded by intensive cultivation [45], and the second-highest human population density of all 21 LCU PAs surveyed (Table S3). As a consequence, Yankari's lions and indeed all large-medium mammals are likely to be effectively isolated from neighboring populations in Nigeria (Kainji Lake NP, ca 650 km distant) and Cameroon (Benoué Complex, ca 260 km distant). Drastic interventions, such as fencing the reserve, may be the only solution to safeguard this population [33]. Fencing Yankari could prevent inevitable encroachment by people and livestock, reduce human-lion conflicts at the PA boundary and perhaps reduce penetration of the PA by poachers.

### The state of PAs in West Africa

Of 12 PAs with management assessments, six had no budget for management activities, and where budgets existed they were much lower than required to conserve lion populations effectively [33]. PAs with confirmed or probable lion presence had larger budgets than those with lions absent, and WAP had both the largest lion population and the highest annual budget, at US$197/km^2^ (Table S4). WAP furthermore consistently received among the highest scores for management effectiveness of all PAs harboring lions (Fig. 4B). For PAs where data on population trends of principal lion prey species were available, WAP (represented through Pendjari NP, where annual wildlife counts are conducted) was also the only site where wildlife numbers were stable or increasing (Fig. 5). As a further indication of conservation success in WAP, this site harbors by far the largest remaining elephant population in West Africa [16], and one of the last remaining populations of the critically endangered Northwest African cheetah (*Acinonyx jubatus ssp. hecki*) [17]. Even so, WAP's budget is an order of magnitude below the estimated >US$2000/km^2^ budget required to maintain lions in unfenced PAs [33], suggesting that WAPs success may not be sustainable and will require increased funding in future.

Staff numbers for LCU PAs were generally low, varying between 0–4 staff per 100 km^2^. Many PAs reported that staff salaries were paid directly by national government and not through the individual PA management budget. Where staff are paid by central government, yet few or no funds are available for active management of PAs, the reported staff numbers likely overestimate conservation effort: nine of the 12 PAs assessed using METT reported having either no law enforcement activity at all (four PAs), or major deficiencies in staff capacity/resources to effect patrols (five PAs). Assessors for Comoé NP, which has 54 patrol staff, commented that *“Staffing is very low for the size of the park; only 2 of 5 sectors of the park are operational, with 1 vehicle, limited staff, no equipment and lack of training”*. In Haut Niger, which has 15 patrol staff, assessors noted that in practice, agents do no or very few patrols due to a lack of resources and motivation; as a result poaching and illegal logging is widespread. Brugière [46] notes that existing PAs in Guinea are essentially paper-parks, i.e. they have no staff, management plan or operating budget. Consequently, even in the largest formally gazetted PA in Guinea, the Kankan Faunal Reserve, where lions are potentially still present, ca 20,000 people live within the PA, poaching pressure is high, and antelope population densities are extremely low [47].

WAP represents the last stronghold for lions in West Africa. Conservation interventions in WAP are heavily subsidized by large international funding bodies, such as the European Union and the World Bank. However, overall investment in conservation activities is extremely low in West Africa, compared to Central, Eastern and Southern Africa [48]. Considering that all eleven former or current lion range countries in West Africa are among the 50 poorest countries in the world, and that six are classified as least developed countries (data from World Bank; http://databank.worldbank.org/data/home.aspx), lion range states in West Africa will be unable to mobilize the resources required to secure their remaining lion populations. That will rely on the provision of substantial financial and technical assistance to range states, principally by the international community, to increase management effectiveness of PAs with lions. For any such investments, it will be imperative that a) conservation initiatives assure sound governance over the funds [49], and b) adequate funding levels are sustained in the long-term to achieve desired outcomes; a review of best practices can be found in Blom et al. [50].

It is imperative to address very widespread poaching of lion prey species and illegal killing of lions by pastoralists within and around PAs [14], [51], [52]. We believe urgent priority must be given to a dual strategy that focuses on 1. increasing the numbers, expertise, and operating budgets of enforcement personnel in PAs with lions, to curb the killing of lion prey and illegal incursions into PAs by pastoralists, and 2. reducing human-lion conflict in affected communities bordering PAs, by combining improved husbandry practices with community sensitization, to reduce livestock losses to predators and ameliorate local negative perceptions of large carnivores [51]. Investment should also be directed toward developing and enhancing photographic tourism in politically stable countries such as Benin and Senegal. This will help to create and maintain economic incentives for lion conservation, and develop enduring revenue streams for PA management not wholly reliant on donor funding.

## Conclusions & Recommendations

The situation for the lion in West Africa is dire. We recommend urgent revision of lion taxonomy by the Cat Classification Task Force of the IUCN/SSC Cat Specialist Group [53]. Recognition of a West-Central African sub-species is supported by recent findings establishing the principal division of extant lions within Africa, and would correctly recognize the genetic uniqueness of West African populations [8], [9]. Irrespective of taxonomic status, we recommend listing the lion as critically endangered in West Africa.

Considering the relative poverty of lion range states in West Africa, we call for the mobilization of substantial and urgent investment by the international community to assist these countries in improving management effectiveness of PAs containing lions. Lions persist in some of the largest and most intact protected landscapes in West Africa, where they co-occur with some of the last remaining populations of critically endangered mammals including Northwest African cheetahs, Western giant elands and African wild dogs. Further deterioration of those last wilderness areas in West Africa will likely cause the loss of genetically distinct populations of charismatic megafauna and further preclude already tenuous, potential future revenue streams from photographic tourism for West African nations. Without immediate action, we believe the opportunity to save both will be lost.

## Supporting Information

Supporting Information S1 The METT tracking tool – a template of the METT questionnaire.
(ZIP)

Table S1 METT (Management Effectiveness Tracking Tool) questions used to evaluate management performance of LCU PAs.
(DOCX)

Table S2 PA management variables used in this study, with sample sizes (number of PAs).
(DOCX)

Table S3 PA attributes for the 21 PAs in this study and details on the field surveys, regarding survey methods and effort, and findings regarding the presence of lions and other species of conservation concern.
(XLSX)

Table S4 PA METT data and scores for the 12 PAs with METT data in this study (PAs labeled using PA number: see S4 for corresponding PA names).
(XLSX)

## References

[1] Barnett R, Shapiro B, Barnes I, Ho SYW, Burger J, et al (2009) Phylogeography of lions (Panthera leo ssp.) reveals three distinct taxa and a late Pleistocene reduction in genetic diversity. Molecular Ecology 18: 1668–1677. 19302360 10.1111/j.1365-294X.2009.04134.x

[2] Nowell K, Jackson P (1996) Wild Cats - Status Survey and Conservation Action Plan. GlandSwitzerland: IUCN/SSC Cat Specialist Group. 421 p.

[3] IUCN (2012) Panthera leo. In: IUCN 2012. IUCN Red List of Threatened Species. Version 2012.2. Available: www.iucnredlist.org.

[4] Riggio J, Jacobson A, Dollar L, Bauer H, Becker M, et al (2013) The size of savannah Africa: a lion's (Panthera leo) view. Biodiversity and Conservation 22: 17–35.

[5] Bauer H, Nowell K (2004) Endangered classification for West African lions. Cat News 41: 35–36.

[6] Bertola LD, van Hooft WF, Vrieling K, Uit de Weerd DR, York DS, et al (2011) Genetic diversity, evolutionary history and implications for conservation of the lion (Panthera leo) in West and Central Africa. Journal of Biogeography 38: 1356–1367.

[7] Mazák JH (2010) Geographical variation and phylogenetics of modern lions based on craniometric data. Journal of Zoology 281: 194–209.

[8] Barnett R, Yamaguchi N, Barnes I, Cooper A (2006) The origin, current diversity and future conservation of the modern lion (*Panthera leo*). Proceedings of the Royal Society B-Biological Sciences 273: 2119–2125. 10.1098/rspb.2006.3555 PMC1635511 16901830

[9] Dubach JM, Briggs MB, White PA, Ament BA, Patterson BD (2013) Genetic perspectives on “Lion Conservation Units” in Eastern and Southern Africa. Conservation Genetics 1–15.

[10] Coad LM, Leverington F, Burgess N, Cuadros I, Geldmann J, et al (2013) Progress towards the CBD protected area management effectiveness targets. PARKS 19: no–no.

[11] Bauer H, De Iongh HH, Princee FP, Ngantou D (2003) Research needs for lion conservation in West and Central Africa. C R Biol 326: 112–118. 10.1016/s1631-0691(03)00047-7 14558459

[12] Craigie ID, Baillie JEM, Balmford A, Carbone C, Collen B, et al (2010) Large mammal population declines in Africa's protected areas. Biological Conservation 143: 2221–2228.

[13] Brashares JS, Arcese P, Sam MK, Coppolillo PB, Sinclair ARE, et al (2004) Bushmeat hunting, wildlife declines, and fish supply in West Africa. Science 306: 1180–1183. 15539602 10.1126/science.1102425

[14] Lindsey PA, Balme G, Becker M, Begg C, Bento C, et al (2013) The bushmeat trade in African savannas: Impacts, drivers, and possible solutions. Biological Conservation 160: 80–96.

[15] Macdonald DW, Johnson PJ, Albrechtsen L, Seymour S, Dupain J, et al (2012) Bushmeat trade in the Cross–Sanaga rivers region: Evidence for the importance of protected areas. Biological Conservation 147: 107–114.

[16] Bouché P, Douglas-Hamilton I, Wittemyer G, Nianogo AJ, Doucet J-L, et al (2011) Will Elephants Soon Disappear from West African Savannahs? PLoS ONE 6: e20619. 21731620 10.1371/journal.pone.0020619 PMC3120750

[17] Belbachir F (2008) Acinonyx jubatus ssp. hecki. In: IUCN 2011. IUCN Red List of Threatened Species. Version 2011.2. Available: www.iucnredlist.org.

[18] IUCN (2006) Conservation strategy for the lion in West and Central Africa. Gland, Switzerland and Cambridge, UK: IUCN SSC Cat Specialist Group.

[19] Sanderson EW, Redford KH, Chetkiewicz CLB, Medellin RA, Rabinowitz AR, et al (2002) Planning to save a species: the jaguar as a model. Conservation Biology 16: 58–72. 10.1046/j.1523-1739.2002.00352.x 35701976

[20] Bauer H, van der Merwe S (2004) Inventory of free-ranging lions *Panthera leo* in Africa. Oryx 38: 26–31.

[21] Brashares JS, Arcese P, Sam MK (2001) Human demography and reserve size predict wildlife extinction in West Africa. Proced Roy Soc Lond B 268: 2473–2478. 10.1098/rspb.2001.1815 PMC1088902 11747566

[22] Woodroffe R, Ginsberg J (1998) Edge effects and the extinction of populations inside protected areas. Science 280: 2126–2128. 9641920 10.1126/science.280.5372.2126

[23] Burton AC (2012) Critical evaluation of a long-term, locally-based wildlife monitoring program in West Africa. Biodiversity and Conservation 21: 3079–3094.

[24] Gros PM (1998) Status of the cheetah in Kenya: a field-interview assessment. Biological Conservation 85: 137–149.

[25] Pettorelli N, Lobora AL, Msuha MJ, Foley C, Durant SM (2010) Carnivore biodiversity in Tanzania: revealing the distribution patterns of secretive mammals using camera traps. Animal Conservation 13: 131–139.

[26] Thorn M, Green M, Bateman PW, Cameron EZ, Yarnell RW, et al (2010) Comparative efficacy of sign surveys, spotlighting and audio playbacks in a landscape-scale carnivore survey. South African Journal of Wildlife Research 40: 77–86.

[27] Stuart C, Stuart T (2003) A field guide to the tracks and signs of Southern and East African wildlife. Cape Town: Struik Publishers.

[28] Funston PJ, Frank L, Stephens T, Davidson Z, Loveridge A, et al (2010) Substrate and species constraints on the use of track incidences to estimate African large carnivore abundance. Journal of Zoology 281: 56–65.

[29] Burton AC, Sam MK, Kpelle DG, Balangtaa C, Buedi EB, et al (2011) Evaluating persistence and its predictors in a West African carnivore community. Biological Conservation 144: 2344–2353.

[30] Ogutu JO, Dublin HT (1998) The response of lions and spotted hyaenas to sound playbacks as a technique for estimating population size. African Journal of Ecology 36: 83–95.

[31] Ferreira SM, Funston PJ (2010) Estimating lion population variables: prey and disease effects in Kruger National Park, South Africa. Wildlife Research 37: 194–206.

[32] Woodroffe R (2000) Predators and people: using human densities to interpret declines of large carnivores. Animal Conservation 3: 165–173.

[33] Packer C, Loveridge A, Canney S, Caro T, Garnett ST, et al (2013) Conserving large carnivores: dollars and fence. Ecology Letters 16: 635–641. 23461543 10.1111/ele.12091

[34] Leverington F, Costa KL, Courrau J, Pavese H, Nolte C, et al.. (2010) Management effectiveness evaluation in protected areas: a global study. Second edition. St Lucia, Queensland, Australia: University of Queensland, IUCN- WCPA, TNC, WWF.

[35] R Core Team (2013) R: A language and environment for statistical computing. R Foundation for Statistical Computing. Vienna, Austria. ISBN 3-900051-07-0. Available: http://www.R-project.org/.

[36] Nyanganji G, Saidu Y, Henschel P, Dunn A (2011) Survey of lions (*Panthera leo*) in Yankari Game Reserve and Kainji Lake National Park, Nigeria. Abuja, Nigeria: Panthera/Wildlife Conservation Society-Nigeria

[37] Stander P (1991) Demography of lions in the Etosha national park, Namibia. Madoqua 18: 1–9.

[38] Schaller GB (1972) The Serengeti lion. Chicago: University of Chicago Press.

[39] IUCN/SSC (2001) IUCN Red List categories and criteria: version 3.1. Prepared by the IUCN Species Survival Commission

[40] Newmark WD (2008) Isolation of African protected areas. Frontiers in Ecology and the Environment 6: 321–328.

[41] East R (1984) Rainfall, soil nutrient status and biomass of large African savanna mammals. African Journal of Ecology 22: 245–270.

[42] Bouché P (2012) Inventaire aérien de l'écosystème W-Arly-Pendjari, Mai–Juin 2012. Ouagadougou, Burkina Faso: CITES-MIKE, WAP/UNOPS, Benin, Burkina Faso, Niger.

[43] IUCN/SSC Antelope Specialist Group (2008) Tragelaphus derbianus ssp. derbianus. In: IUCN 2012. IUCN Red List of Threatened Species. Version 2012.2. Available: www.iucnredlist.org.

[44] Woodroffe R, Sillero-Zubiri C (2012) *Lycaon pictus* (West Africa subpopulation). In: IUCN 2013. IUCN Red List of Threatened Species. Version 2013.1. Available: www.iucnredlist.org.

[45] Inah EI, Orimoyegun SO (2005) Herbaceous cover and soil properties in wildlife habitats in Bauchi and Gombe States, Nigeria. Journal of Sustainable Agriculture 27: 125–136.

[46] Brugière D (2012) Identifying priority areas for the conservation of antelopes in the Republic of Guinea, West Africa, using the complementarity approach. Oryx 46: 253–259.

[47] Dufour S (2008) Programme de gestion communautaire et participative de la faune sauvage dans la Réserve de Faune de Kankan, République de Guinée. Conakry, Guinea: Rapport de mission. Sylvatrop.

[48] Brockington D, Scholfield K (2010) Expenditure by conservation nongovernmental organizations in sub-Saharan Africa. Conservation Letters 3: 106–113.

[49] Smith R, Muir R, Walpole M, Balmford A, Leader-Williams N (2003) Governance and the loss of biodiversity. Nature 426: 67–70. 14603318 10.1038/nature02025

[50] Blom B, Sunderland T, Murdiyarso D (2010) Getting REDD to work locally: lessons learned from integrated conservation and development projects. Environmental Science & Policy 13: 164–172.

[51] Bauer H, de Iongh H, Sogbohossou E (2010) Assessment and mitigation of human-lion conflict in West and Central Africa. Mammalia 74: 363–367.

[52] Henschel P, Azani D, Burton C, Malanda G, Saidu Y, et al (2010) Lion status updates from five range countries in West and Central Africa. Cat News 52: 34–39.

[53] Breitenmoser U, Breitenmoser C, Nowell K (2012) The next Red List assessment of the cats. Cat News 57: 3.

[54] Granier N, Martinez L (2004) Etude des Chimpanzés de l'Aire Protégée «Bafing-Falémé»: Enquêtes auprès des Populations Locales et Dénombrement par comptage de Nids. Bamako, Mali: EU Delegation, Republic of Mali.

[55] PFNH (2009) Rapport d'activités Mai–Juillet 2009. Conakry, Guinea: Projet d'Etude des Grands Félins et de leur Environnement Naturel et Humain.

[56] Delvingt W, Vermeulen C (2007) Nazinga. Gembloux: Presses Agronomiques de Gembloux.

[57] Bouché P, Lungren CG, Hien B, Omondi P (2004) Recensement aérien total de l'Ecosystème W-Arly-Pendjari-Oti-Mandouri-Kéran (WAPOK). CITES-MIKE, ECOPAS, PAUCOF, Benin, Burkina Faso, Niger, Togo. Available: http://www.cites.org/eng/prog/MIKE/index.shtml.

[58] CENAGREF (2013) Plan d'action pour la conservation du lion au Bénin. Cotonou, République du Bénin: CENAGREF.

[59] Galat G, Benoit M, Chevillotte H, Diop A, Duplantier J-M, et al.. (1992) Dénombrement de la grande faune du Parc National du Niokolo-Koba, Sénégal, 1990–1991. Dakar: Direction des Parcs Nationaux du Senegal.

[60] Sinsin B, Tehou A, Daouda I, Saidou A (2002) Abundance and species richness of larger mammals in Pendjari National Park in Benin. Mammalia 66: 369–380.

[61] WD (2005) Mole National Park Management Plan. WDSP Report No 54. Accra, Ghana: Wildlife Division of Ghana, Government of Ghana.

[62] Bouché P (2006) Mole wildlife survey. Accra, Ghana: Northern Savannah Biodiversity Conservation Project/IUCN.

[63] Renaud P-C, Gueye MB, Hejcmanová P, Antoninova M, Samb M (2006) Inventaire aérien et terrestre de la faune et relevé des pressions au Parc National du Niokolo Koba. African Parks Network

