# Supplementary material for: The Lion in West Africa Is Critically Endangered
Source: PLoS One. 2014 Jan 8;9(1):e83500. doi: 10.1371/journal.pone.0083500 (PMC3885426; doi:10.1371/journal.pone.0083500)
Supplement: Supporting Information S1 — The METT tracking tool – a template of the METT questionnaire. (ZIP) [file pone.0083500.s001.zip › Henschel et al_S1.pdf]

WWF

The World Bank

Reporting Progress at Protected Area Sites

A simple site-level tracking tool developed for the World Bank and WWF

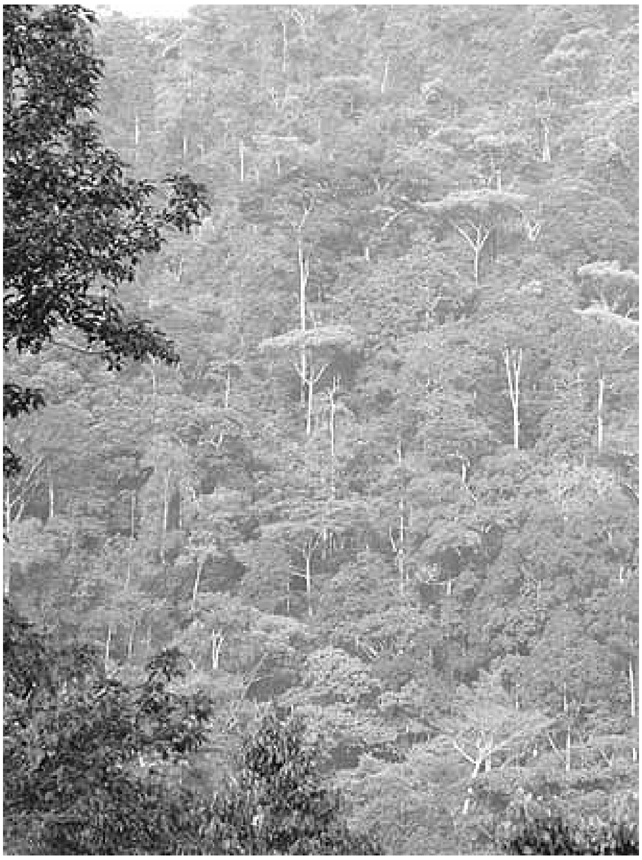

Prepared for the World Bank/WWF Forest Alliance

Contents

Background	3
The WCPA Framework	4
Purpose of the World Bank/WWF Management Effectiveness Tracking Tool	5
Guidance notes for using the World Bank/WWF Management Effectiveness Tracking Tool	6
Data Sheet and Questionnaire	7

Cover photograph of Bwindi Impenetrable Forest Reserve, Uganda by Marc Hockings

Many thanks to those people who commented on earlier drafts, including Rod Atkins, David Cassells, Peter Cochrane, Finn Danielsen, Jamison Ervin, Jack Hurd, Glenys Jones, Leonardo Lacerda, Rosa Lemos de Sá, Mariana Montoya, Marianne Meijboom, Sheila O'Connor, Christian Peter, Jeff Sayer. This version of the system also benefited considerably from a consultant's report written by Antoine Leclerc, who interviewed many people in WWF's Indochina Programme about the tracking tool and their experience is reflected here.

Sue Stolton, Marc Hockings, Nigel Dudley, Kathy MacKinnon and Tony Whitten

April 2003, with adaptations in 2005

Background

There is a growing concern amongst protected area professionals that many protected areas around the world are not achieving the objectives for which they were established. One response to this concern has been an emphasis on the need to increase the effectiveness of protected area management, and to help this process a number of assessment tools have been developed to assess management practices. It is clear that the existence of a wide range of situations and needs require different methods of assessment. The World Commission on Protected Areas (WCPA) has therefore developed a 'framework' for assessment[1]. The WCPA framework aims both to provide some overall guidance in the development of assessment systems and to encourage standards for assessment and reporting.

The WCPA Framework is based on the idea that good protected area management follows a process that has six distinct stages, or elements:

- it begins with understanding the **context** of existing values and threats,
- progresses through **planning**, and
- allocation of resources (**inputs**), and
- as a result of management actions (**processes**),
- eventually produces products and services (**outputs**),
- that result in impacts or **outcomes**.

The World Bank/WWF Alliance for Forest Conservation and Sustainable Use ('the Alliance') was formed in April 1998, in response to the continued depletion of the world's forest biodiversity and of forest-based goods and services essential for sustainable development. As part of its programme of work the Alliance has set a target relating to management effectiveness of protected areas: *50 million hectares of existing but highly threatened forest protected areas to be secured under effective management by the year 2005*[2]*.*To evaluate progress towards this target the Alliance has developed a simple site-level tracking tool to facilitate reporting on management effectiveness of protected areas within WWF and World Bank projects. The tracking tool has been built around the application of the WCPA Framework and Appendix II of the Framework document has provided its basic structure.

The World Bank/WWF Management Effectiveness Tracking Tool forms part of a series of management effectiveness assessment tools, which range from the *WWF Rapid Assessment and Prioritisation Methodology* used to identify key protected areas at threat within a protected area system to detailed monitoring systems such as those being developed by the *Enhancing Our Heritage* project for UNESCO natural World Heritage sites. The Alliance has also supported the development of both the WCPA framework and the development of the WWF Rapid Assessment and Prioritisation Methodology.

---

[1] Hockings, Marc with Sue Stolton and Nigel Dudley (2000); *Assessing Effectiveness – A Framework for Assessing Management Effectiveness of Protected Areas*; University of Cardiff and IUCN, Switzerland
[2] Dudley, Nigel and Sue Stolton (1999); *Threats to Forest Protected Areas: Summary of a survey of 10 countries*; project carried out for the WWF/World Bank Alliance in association with the IUCN World Commission on Protected Areas, IUCN, Switzerland

The WCPA Framework

To maximise the potential of protected areas, and to improve management processes, we need to understand the strengths and weaknesses of their management and the threats that they face. In the last few years, various methodologies for assessing management effectiveness of protected areas have been developed and tested around the world. The World Commission on Protected Areas provides an overarching framework for assessing management effectiveness of both protected areas and protected area systems, to give guidance to managers and others and to help harmonise assessment around the world.

Table 1 contains a very brief summary of the elements of the WCPA Framework and the criteria that can be assessed[3]. The World Bank/WWF Management Effectiveness Tracking Tool has been designed to fulfil the elements of evaluation included in the Framework.

Table 1: Summary of the WCPA Framework

Elements of evaluation	Explanation	Criteria that are assessed	Focus of evaluation
**Context**	***Where are we now?*** Assessment of importance, threats and policy environment	- Significance<br>- Threats<br>- Vulnerability<br>- National context<br>- Partners	Status
**Planning**	***Where do we want to be?*** Assessment of protected area design and planning	- Protected area legislation and policy<br>- Protected area system design<br>- Reserve design<br>- Management planning	Appropriateness
**Inputs**	***What do we need?*** Assessment of resources needed to carry out management	- Resourcing of agency<br>- Resourcing of site	Resources
**Processes**	***How do we go about it?*** Assessment of the way in which management is conducted	- Suitability of management processes	Efficiency and appropriateness
**Outputs**	***What were the results?*** Assessment of the implementation of management programmes and actions; delivery of products and services	- Results of management actions<br>- Services and products	Effectiveness
**Outcomes**	***What did we achieve?*** Assessment of the outcomes and the extent to which they achieved objectives	- Impacts: effects of management in relation to objectives	Effectiveness and appropriateness

Questions in the following tracking tool have been ordered to make completion as easy as possible; the element(s) that each refers to are indicated in the left hand column.

[3] For a copy of the WPCA Framework or a more detailed summary please visit the WCPA web-site at: www.iucn.org/themes/wcpa or contact WCPA at wcpa@hq.iucn.org

Purpose of the World Bank/WWF Management Effectiveness Tracking Tool

The World Bank/WWF Management Effectiveness Tracking Tool has been developed to help track and monitor progress in the achievement of the World Bank/WWF Alliance worldwide protected area management effectiveness target. It is also hoped that the tracking tool will be used more generally where it can help monitor progress towards improving management effectiveness; for example it is being used by the Global Environment Facility.

The Alliance has identified that the tracking tool needs to be:

- Capable of providing a harmonised reporting system for protected area assessment within both the World Bank and WWF;
- Suitable for replication;
- Able to supply consistent data to allow tracking of progress over time;
- Relatively quick and easy to complete by protected area staff, so as not to be reliant on high levels of funding or other resources;
- Capable of providing a “score” if required;
- Based around a system that provides four alternative text answers to each question, strengthening the scoring system;
- Easily understood by non-specialists; and
- Nested within existing reporting systems to avoid duplication of effort.

Limitations

The World Bank/WWF Management Effectiveness Tracking Tool is aimed to help ***reporting progress*** on management effectiveness and should not replace more thorough methods of assessment for the purposes of adaptive management. The tracking tool has been developed to provide a quick overview of progress in improving the effectiveness of management in individual protected areas, to be filled in by the protected area manager or other relevant site staff. As such it is clear that there are strict limitations on what it can achieve: it should not for example be regarded as an independent assessment, or as the sole basis for adaptive management.

Because of the great differences between expectations, resources and needs around the world, the tracking tool also has strict limitations in terms of allowing comparison between sites: the scoring system, if applied at all, will be most useful for tracking progress over time in one site or a closely related group of sites.

Lastly, the tracking tool is too limited to allow a detailed evaluation of *outcomes* and is really aimed at providing a quick overview of the management steps identified in the WCPA Framework up to and including *outputs*. Although we include some questions relating to outcomes, the limitations of these should be noted. Clearly, however good management is, if biodiversity continues to decline, the protected area objectives are not being met. Therefore the question on condition assessment has disproportionate importance in the overall tracking tool.

Guidance notes for using the Tracking Tool

The World Bank/WWF Management Effectiveness Tracking Tool can be completed by protected area staff or project staff, with input from other protected area staff. The tracking tool has been designed to be easily answered by those managing the protected area without any additional research.

All sections of the tracking tool should be completed. There are two sections:

1. **Datasheet**: which details key information on the site, its characteristics and management objectives and includes an overview of WWF/World Bank involvement.

2. **Assessment Form**: the assessment form includes three distinct sections, all of which should be completed.

   - Questions and scores: the main part of the assessment form is a series of 30 questions that can be answered by ***assigning a simple score ranging between 0 (poor) to 3 (excellent)***. A series of four alternative answers are provided against each question to help assessors to make judgements as to the level of score given. ***Questions that are not relevant to a particular protected area should be omitted***, with a reason given in the comments section (for example questions about use and visitors will not be relevant to a protected area managed according to the IUCN protected area management Category Ia). In addition, there are six supplementary questions which elaborate on key themes in the previous questions and provide additional information and points. This is, inevitably, an approximate process and there will be situations in which none of the four alternative answers appear to fit conditions in the protected area very precisely. We suggest that you choose the answer that is nearest and use the comments section to elaborate.

   - Comments: a box next to each question allows for ***qualitative judgements to be justified*** by explaining why they were made (this could range from personal opinion, a reference document, monitoring results or external studies and assessments – the point being to give anyone reading the report an idea of why the assessment was made). In this section we also suggest that respondents comment on the role/influence of WWF or World Bank projects if appropriate. On some occasions suggestions are made about what might be covered in the comments column.

   - Next Steps: for each question respondents are asked to identify a long-term management need to further adaptive management at the site, if this is relevant.

3. **Final Score**: a final total of the score from completing the assessment form can be ***calculated as a percentage of scores from those questions that were relevant to a particular protected area***. (So for example if 5 questions are believed to be irrelevant (and this is justified in the comments column) then the final score would be multiplied by 30/25 to offset the fact that some questions were not applied.) If the additional questions are relevant to the protected area, add the additional score to the total if they are relevant and omit them if they are not.

Disclaimer: The whole concept of “scoring” progress is fraught with difficulties and possibilities for distortion. The current system assumes, for example, that all the questions cover issues of equal weight, whereas this is not necessarily the case. Accuracy might be improved by weighting the various scores although this would provide additional challenges in deciding differing weightings. In the current version a simple scoring system is maintained, but the limitations of this approach should be recognised.

Reporting Progress at Protected Area Sites: Data Sheet

Name of protected area		
Location of protected area (country, province, and if possible map reference)		
Is it located in G200 ecoregion? If yes, provide G200 name and number	☐ No	☐ Yes
Date of establishment		
Ownership details (i.e. owner, tenure rights etc)		
Management Authority		
Size of protected area (ha)		
Number of staff	Permanent	Temporary
Annual budget (US$)		
Designations (IUCN category, World Heritage, Ramsar etc)		
Reasons for designation		
Brief details of World Bank funded project or projects in PA		
Brief details of WWF funded project or projects in PA		
Brief details of other relevant projects in PA		
List the two primary protected area objectives		
Objective 1		
Objective 2		

Name/s of assessor (including people consulted):

____

Contact details (email etc.): ____

____

Date assessment carried out: ____

List the top two most important threats to the PA values<br>(please tick **ONLY TWO** boxes on this page)	Indicate reasons why these were chosen
1 Habitat conversion:<br>☐ housing<br>☐ industrial development<br>☐ farms<br>☐ plantations<br>☐ ski areas<br>☐ dams<br>☐ other / non specified<br><br>2 Transportation/Energy infrastructure:<br>☐ utility lines<br>☐ roads<br>☐ railroads<br>☐ wind farms<br>☐ other / non specified<br><br>3 Abiotic resource use:<br>☐ mining<br>☐ oil & gas drilling<br>☐ geothermal energy<br>☐ water withdrawal<br>☐ other / non specified<br><br>4 Consumptive biological resource use:<br>☐ hunting<br>☐ NTFP collection<br>☐ grazing<br>☐ logging<br>☐ other / non specified<br><br>5 Non-consumptive biological resource use:<br>☐ ATVs/snowmobiles<br>☐ hiking/biking<br>☐ scientific research<br>☐ military maneuvers<br>☐ other / non specified<br><br>6 Pollution:<br>☐ acid rain<br>☐ solid waste<br>☐ toxins<br>☐ radio active fallout<br>☐ other / non specified<br><br>7 Invasive species (alien and native):<br>☐ plants<br>☐ animals<br>☐ disease & pathogens<br>☐ other / non specified<br><br>8 Modification of natural processes / ecological drivers / disturbance regimes:<br>☐ climate change<br>☐ loss of key predators<br>☐ grazing patterns<br>☐ fire regime<br>☐ fragmentation<br>☐ other / non specified<br><br>9 Other<br>☐ other / non specified	

List top two critical management activities where PA staff is investing most of their time (please tick **ONLY TWO** boxes on this page)	Comments
☐ Law enforcement and surveillance<br>☐ Promoting sustainable resource use<br>☐ Working with local communities<br>☐ Education and awareness<br>☐ Demarcation and zoning<br>☐ Monitoring<br>☐ Research<br>☐ Management planning<br>☐ Ecotourism<br>☐ Building institutional and governance capacity<br>☐ Species management<br>☐ Infrastructure development<br>☐ Fundraising<br>☐ Restoration<br>☐ Fire management<br>☐ Resolving tenure problems<br>☐ Human-wildlife conflict management<br>☐ Alien species control<br>☐ External communication and Publicity<br>☐ Equipment & facilities<br>☐ Improving habitat<br>☐ Species (re)introduction / control / breeding<br>☐ Working with regional authorities<br>☐ Other	

Issue	Criteria	Score	Comments	Next steps
1. Legal status<br><br>Does the protected area have legal status?<br><br>*Context*	The protected area is not gazetted	0	*Note*: see fourth option for private reserves	
	The government has agreed that the protected area should be gazetted but the process has not yet begun	1		
	The protected area is in the process of being gazetted but the process is still incomplete	2		
	The protected area has been legally gazetted (or in the case of private reserves is owned by a trust or similar)	3		
2. Protected area regulations<br><br>Are inappropriate land uses and activities (e.g. poaching) controlled?<br><br>*Context*	There are no mechanisms for controlling inappropriate land use and activities in the protected area	0		
	Mechanisms for controlling inappropriate land use and activities in the protected area exist but there are major problems in implementing them effectively	1		
	Mechanisms for controlling inappropriate land use and activities in the protected area exist but there are some problems in effectively implementing them	2		
	Mechanisms for controlling inappropriate land use and activities in the protected area exist and are being effectively implemented	3		
3. Law enforcement<br><br>Can staff enforce protected area rules well enough?<br><br>*Context*	The staff have no effective capacity/resources to enforce protected area legislation and regulations	0	*Possible issue for comment*: What happens if people are arrested?	
	There are major deficiencies in staff capacity/resources to enforce protected area legislation and regulations (e.g. lack of skills, no patrol budget)	1		
	The staff have acceptable capacity/resources to enforce protected area legislation and regulations but some deficiencies remain	2		
	The staff have excellent capacity/resources to enforce protected area legislation and regulations	3		
4. Protected area objectives<br><br>Have objectives been agreed?<br><br>*Planning*	No firm objectives have been agreed for the protected area	0		
	The protected area has agreed objectives, but is not managed according to these objectives	1		
	The protected area has agreed objectives, but these are only partially implemented	2		
	The protected area has agreed objectives and is managed to meet these objectives	3		

Issue	Criteria	Score	Comments	Next steps
5. Protected area design<br><br>Does the protected area need enlarging, corridors etc to meet its objectives?<br><br>*Planning*	Inadequacies in design mean achieving the protected areas major management objectives of the protected area is impossible	0	*Possible issue for comment*: does the protected area contain different management zones and are these well maintained?	
	Inadequacies in design mean that achievement of major objectives are constrained to some extent	1		
	Design is not significantly constraining achievement of major objectives, but could be improved	2		
	Reserve design features are particularly aiding achievement of major objectives of the protected area	3		
6. Protected area boundary demarcation<br><br>Is the boundary known and demarcated?<br><br>*Context*	The boundary of the protected area is not known by the management authority or local residents/neighbouring land users	0	*Possible issue for comment*: are there tenure disagreements affecting the protected area?	
	The boundary of the protected area is known by the management authority but is not known by local residents/neighbouring land users	1		
	The boundary of the protected area is known by both the management authority and local residents but is not appropriately demarcated	2		
	The boundary of the protected area is known by the management authority and local residents and is appropriately demarcated	3		
7. Management plan<br><br>Is there a management plan and is it being implemented?<br><br>*Planning*	There is no management plan for the protected area	0		
	A management plan is being prepared or has been prepared but is not being implemented	1		
	An approved management plan exists but it is only being partially implemented because of funding constraints or other problems	2		
	An approved management plan exists and is being implemented	3		
Additional points<br><br>*Planning*	The planning process allows adequate opportunity for key stakeholders to influence the management plan	+1		
	There is an established schedule and process for periodic review and updating of the management plan	+1		
	The results of monitoring, research and evaluation are routinely incorporated into planning	+1		

Issue	Criteria	Score	Comments	Next steps
8. Regular work plan<br><br>Is there an annual work plan?<br><br>*Planning/Outputs*	No regular work plan exists	0		
	A regular work plan exists but activities are not monitored against the plan's targets	1		
	A regular work plan exists and actions are monitored against the plan's targets, but many activities are not completed	2		
	A regular work plan exists, actions are monitored against the plan's targets and most or all prescribed activities are completed	3		
9. Resource inventory<br><br>Do you have enough information to manage the area?<br><br>*Context*	There is little or no information available on the critical habitats, species and cultural values of the protected area	0		
	Information on the critical habitats, species and cultural values of the protected area is not sufficient to support planning and decision making	1		
	Information on the critical habitats, species and cultural values of the protected area is sufficient for key areas of planning/decision making but the necessary survey work is not being maintained	2		
	Information concerning on the critical habitats, species and cultural values of the protected area is sufficient to support planning and decision making and is being maintained	3		
10. Research<br><br>Is there a programme of management-orientated survey and research work?<br><br>*Inputs*	There is no survey or research work taking place in the protected area	0		
	There is some *ad hoc* survey and research work	1		
	There is considerable survey and research work but it is not directed towards the needs of protected area management	2		
	There is a comprehensive, integrated programme of survey and research work, which is relevant to management needs	3		
11. Resource management<br><br>Is the protected area adequately managed (e.g. for fire, invasive species, poaching)?<br><br>*Process*	Requirements for active management of critical ecosystems, species and cultural values have not been assessed	0		
	Requirements for active management of critical ecosystems, species and cultural values are known but are not being addressed	1		
	Requirements for active management of critical ecosystems, species and cultural values are only being partially addressed	2		
	Requirements for active management of critical ecosystems, species and cultural values are being substantially or fully addressed	3		

Issue	Criteria	Score	Comments	Next steps
12. Staff numbers<br><br>Are there enough people employed to manage the protected area?<br><br>*Inputs*	There are no staff	0		
	Staff numbers are inadequate for critical management activities	1		
	Staff numbers are below optimum level for critical management activities	2		
	Staff numbers are adequate for the management needs of the site	3		
13. Personnel management<br><br>Are the staff managed well enough?<br><br>*Process*	Problems with personnel management constrain the achievement of major management objectives	0		
	Problems with personnel management partially constrain the achievement of major management objectives	1		
	Personnel management is adequate to the achievement of major management objectives but could be improved	2		
	Personnel management is excellent and aids the achievement major management objectives	3		
14. Staff training<br><br>Is there enough training for staff?<br><br>*Inputs/Process*	Staff are untrained	0		
	Staff training and skills are low relative to the needs of the protected area	1		
	Staff training and skills are adequate, but could be further improved to fully achieve the objectives of management	2		
	Staff training and skills are in tune with the management needs of the protected area, and with anticipated future needs	3		
15. Current budget<br><br>Is the current budget sufficient?<br><br>*Inputs*	There is no budget for the protected area	0		
	The available budget is inadequate for basic management needs and presents a serious constraint to the capacity to manage	1		
	The available budget is acceptable, but could be further improved to fully achieve effective management	2		
	The available budget is sufficient and meets the full management needs of the protected area	3		

Issue	Criteria	Score	Comments	Next steps
16. Security of budget<br><br>Is the budget secure?<br><br>*Inputs*	There is no secure budget for the protected area and management is wholly reliant on outside or year by year funding	0		
	There is very little secure budget and the protected area could not function adequately without outside funding	1		
	There is a reasonably secure core budget for the protected area but many innovations and initiatives are reliant on outside funding	2		
	There is a secure budget for the protected area and its management needs on a multi-year cycle	3		
17. Management of budget<br><br>Is the budget managed to meet critical management needs?<br><br>*Process*	Budget management is poor and significantly undermines effectiveness	0		
	Budget management is poor and constrains effectiveness	1		
	Budget management is adequate but could be improved	2		
	Budget management is excellent and aids effectiveness	3		
18. Equipment<br><br>Is equipment sufficient?<br><br>*Process*	There is little or no equipment and facilities	0		
	There is some equipment and facilities but these are wholly inadequate	1		
	There is equipment and facilities, but still some major gaps that constrain management	2		
	There is adequate equipment and facilities	3		
19. Maintenance of equipment<br><br>Is equipment adequately maintained?<br><br>*Process*	There is little or no maintenance of equipment and facilities	0		
	There is some *ad hoc* maintenance of equipment and facilities	1		
	There is maintenance of equipment and facilities, but there are some important gaps in maintenance	2		
	Equipment and facilities are well maintained	3		

Issue	Criteria	Score	Comments	Next steps
20. Education and awareness programme<br>Is there a planned education programme?<br><br>*Process*	There is no education and awareness programme	0		
	There is a limited and *ad hoc* education and awareness programme, but no overall planning for this	1		
	There is a planned education and awareness programme but there are still serious gaps	2		
	There is a planned and effective education and awareness programme fully linked to the objectives and needs of the protected area	3		
21. State and commercial neighbours<br>Is there co-operation with adjacent land users?<br><br>*Process*	There is no contact between managers and neighbouring official or corporate land users	0		
	There is limited contact between managers and neighbouring official or corporate land users	1		
	There is regular contact between managers and neighbouring official or corporate land users, but only limited co-operation	2		
	There is regular contact between managers and neighbouring official or corporate land users, and substantial co-operation on management	3		
22. Indigenous people<br><br>Do indigenous and traditional peoples resident or regularly using the PA have input to management decisions?<br>*Process*	Indigenous and traditional peoples have no input into decisions relating to the management of the protected area	0		
	Indigenous and traditional peoples have some input into discussions relating to management but no direct involvement in the resulting decisions	1		
	Indigenous and traditional peoples directly contribute to some decisions relating to management	2		
	Indigenous and traditional peoples directly participate in making decisions relating to management	3		
23. Local communities<br><br>Do local communities resident or near the protected area have input to management decisions?<br>*Process*	Local communities have no input into decisions relating to the management of the protected area	0		
	Local communities have some input into discussions relating to management but no direct involvement in the resulting decisions	1		
	Local communities directly contribute to some decisions relating to management	2		
	Local communities directly participate in making decisions relating to management	3		

Issue	Criteria	Score	Comments	Next steps
Additional points<br><br>*Outputs*	There is open communication and trust between local stakeholders and protected area managers	+1		
	Programmes to enhance local community welfare, while conserving protected area resources, are being implemented	+1		
24. Visitor facilities<br><br>Are visitor facilities (for tourists, pilgrims etc) good enough?<br><br>*Outputs*	There are no visitor facilities and services	0	*Possible issue for comment*: Do visitors damage the protected area?	
	Visitor facilities and services are inappropriate for current levels of visitation or are under construction	1		
	Visitor facilities and services are adequate for current levels of visitation but could be improved	2		
	Visitor facilities and services are excellent for current levels of visitation	3		
25. Commercial tourism<br><br>Do commercial tour operators contribute to protected area management?<br><br>*Process*	There is little or no contact between managers and tourism operators using the protected area	0	*Possible issue for comment*: examples of contributions	
	There is contact between managers and tourism operators but this is largely confined to administrative or regulatory matters	1		
	There is limited co-operation between managers and tourism operators to enhance visitor experiences and maintain protected area values	2		
	There is excellent co-operation between managers and tourism operators to enhance visitor experiences, protect values and resolve conflicts	3		
26. Fees<br>If fees (tourism, fines) are applied, do they help protected area management?<br><br>*Outputs*	Although fees are theoretically applied, they are not collected	0		
	The fee is collected, but it goes straight to central government and is not returned to the protected area or its environs	1		
	The fee is collected, but is disbursed to the local authority rather than the protected area	2		
	There is a fee for visiting the protected area that helps to support this and/or other protected areas	3		
27. Condition assessment<br><br>Is the protected area being managed consistent to its objectives?<br>*Outcomes*	Important biodiversity, ecological and cultural values are being severely degraded	0	*Possible issue for comment:* It is important to provide details of the biodiversity, ecological or cultural values being affected	
	Some biodiversity, ecological and cultural values are being severely degraded	1		
	Some biodiversity, ecological and cultural values are being partially degraded but the most important values have not been significantly impacted	2		
	Biodiversity, ecological and cultural values are predominantly intact	3		

Issue	Criteria	Score	Comments	Next steps
Additional points<br><br>*Outputs*	There are active programmes for restoration of degraded areas within the protected area and/or the protected area buffer zone	+1		
28. Access assessment<br><br>Is access/resource use sufficiently controlled?<br><br>*Outcomes*	Protection systems (patrols, permits etc) are ineffective in controlling access or use of the reserve in accordance with designated objectives	0		
	Protection systems are only partially effective in controlling access or use of the reserve in accordance with designated objectives	1		
	Protection systems are moderately effective in controlling access or use of the reserve in accordance with designated objectives	2		
	Protection systems are largely or wholly effective in controlling access or use of the reserve in accordance with designated objectives	3		
29. Economic benefit assessment<br><br>Is the protected area providing economic benefits to local communities?<br><br>*Outcomes*	The existence of the protected area has reduced the options for economic development of the local communities	0	*Possible issue for comment*: how does national or regional development impact on the protected area?	
	The existence of the protected area has neither damaged nor benefited the local economy	1		
	There is some flow of economic benefits to local communities from the existence of the protected area but this is of minor significance to the regional economy	2		
	There is a significant or major flow of economic benefits to local communities from activities in and around the protected area (e.g. employment of locals, locally operated commercial tours etc)	3		
30. Monitoring and evaluation<br><br>Are management activities monitored against performance?<br><br>*Planning/Process*	There is no monitoring and evaluation in the protected area	0		
	There is some *ad hoc* monitoring and evaluation, but no overall strategy and/or no regular collection of results	1		
	There is an agreed and implemented monitoring and evaluation system but results are not systematically used for management	2		
	A good monitoring and evaluation system exists, is well implemented and used in adaptive management	3		
**TOTAL SCORE**				
