# Supplementary material for: The Lion in West Africa Is Critically Endangered
Source: PLoS One. 2014 Jan 8;9(1):e83500. doi: 10.1371/journal.pone.0083500 (PMC3885426; doi:10.1371/journal.pone.0083500)
Supplement: Table S1 — METT (Management Effectiveness Tracking Tool) questions used to evaluate management performance of LCU PAs. (DOCX) [file pone.0083500.s002.docx]

**Table S1** METT (Management Effectiveness Tracking Tool) questions used to evaluate management performance of LCU PAs.

| PA Management Characteristic | Score | | | |
| --- | --- | --- | --- | --- |
|  | 0 | 1 | 2 | 3 |
| Current Budget | No budget | Inadequate for management; serious constraint | Acceptable but could be improved | Sufficient; meets full management needs |
| Security of Budget | No secure budget; depends on outside/ variable funding | Little secure budget; requires outside funding assistance | Reasonably secure core budget | Secure budget for PA on a multi-year cycle. |
| Fees | No fee collected | Fee dispersed only to central government | Fee dispersed to local authority | Fee helps to support this and/or other PAs |
| Staff | No staff | Inadequate for critical management activities | Below optimum for critical management activities | Adequate for management needs |
| Equipment | No equipment | Equipment wholly inadequate | Major gaps in equipment that constrain management | Adequate equipment |
| Management plan | No plan | In preparation | Partially implemented | Fully implemented |
| Annual work plan | No plan | Few activities implemented | Many activities implemented | All activities implemented |
| Boundary demarcation | Not known | Known by management only | Known by local residents but not demarcated | Known by local residents and demarcated |
| Law enforcement | No capacity | Major deficiencies | Acceptable capacity | Excellent capacity |
| Visitor Facilities | No visitor facilities | Inappropriate facilities/ under construction | Adequate facilities; could be improved | Excellent facilities for current visitation levels |
| Research | No survey or research work | Small amount, not directed towards management needs | Considerable, not directed towards management needs | Comprehensive, directed towards management needs |
| Monitoring and evaluation (M&E) | No monitoring and evaluation | Some ad hoc M&E but no strategy | Agreed M&E strategy; results not used for management | Good M&E system, use for adaptive management |
| Condition assessment | Important values being degraded | Some values being degraded | Most important values not significantly impacted | Values are predominantly intact |
