# Supplementary material for: The Lion in West Africa Is Critically Endangered
Source: PLoS One. 2014 Jan 8;9(1):e83500. doi: 10.1371/journal.pone.0083500 (PMC3885426; doi:10.1371/journal.pone.0083500)
Supplement: Table S2 — PA management variables used in this study, with sample sizes (number of PAs). (DOCX) [file pone.0083500.s003.docx]

**Table S2** PA management variables used in this study, with sample sizes (number of PAs)

| Management Characteristic | Data provenance | Description | Sample size (PAs) |
| --- | --- | --- | --- |
| Non-scored characteristics | | | |
| IUCN category | PP and PA managers | IUCN Protected Area management category was provided by PA managers, or the UNEP-WCMC World Database on Protected Areas (http://www.protectedplanet.net) | 21 |
| Area | PP and PA managers | Area (km^2^) was calculated in ArcGIS, from PA shapefiles downloaded from the UNEP-WCMC World Database on Protected Areas (<http://www.protectedplanet.net>) and corrected, where necessary, based on input by PA managers or researchers | 21 |
| Human population density | Calculated for this study | Human population density around PAs based on human population data from the AfriPop Project ([www.afripop.org](http://www.afripop.org)). A 5km buffer was created around each PA in ArcGIS, and the population density (people/km^2^) within this buffer calculated. | 21 |
| Budget | METT | The Annual PA operating budget (excluding staff salaries) | 12 |
| Permanent staff number | METT | The total number of permanent staff employed by the PA | 12 |
| Patrol Staff number | METT | In many of the PAPACO-led METT evaluations, PA managers provided details of the number of patrol staff employed by the PA. This information was given in the ‘comments’ box for the scoring of ‘Staff Numbers’. | 11  (not available for Oti- Mandouri) |
| Scored characteristics | | | |
| Current Budget | METT | Scored 0 – 3:  0: There is no budget for management of the protected area  1: The available budget is inadequate for basic management needs and presents a serious constraint to the capacity to manage  2: The available budget is acceptable but could be further improved to fully achieve effective management  3: The available budget is sufficient and meets the full management needs of the protected area | 12 |
| Security of Budget | METT | Scored 0 – 3:  0: There is no secure budget for the protected area and management is wholly reliant on outside or year by year funding  1: There is very little secure budget and the protected area could not function adequately without outside funding  2: There is a reasonably secure core budget for the protected area but many innovations and initiatives are reliant on outside funding  3: There is a secure budget for the protected area and its management needs on a multi-year cycle | 11 (answer missing for WAP) |
| Fees | METT | Scored 0 – 3:  0: Although fees are theoretically applied, they are not collected  1: The fee is collected, but it goes straight to central government and is not returned to the protected area or its environs  2: The fee is collected, but is disbursed to the local authority rather than the protected area  3: There is a fee for visiting the protected area that helps to support this and/or other protected areas | 12 |
| Staff numbers | METT | Scored 0 – 3:  0: There are no staff  1: Staff numbers are inadequate for critical management activities  2: Staff numbers are below optimum level for critical management activities  3: Staff numbers are adequate for the management needs of the site | 12 |
| Equipment | METT | Scored 0 – 3:  0: There is little or no equipment and facilities  1: There is some equipment and facilities but these are wholly inadequate  2: There is equipment and facilities, but still some major gaps that constrain management  3: There is adequate equipment and facilities | 12 |
| Management plan | METT | Scored 0 – 3:  0: There is no management plan for the protected area  1: A management plan is being prepared or has been prepared but is not being implemented  2: An approved management plan exists but it is only being partially implemented because of funding constraints or other problems  3: An approved management plan exists and is being implemented | 12 |
| Annual work plan | METT | Scored 0 – 3:  0: No regular work plan exists  1: A regular work plan exists but activities are not monitored against the plan’s targets  2: A regular work plan exists and actions are monitored against the plan’s targets, but many activities are not completed  3: A regular work plan exists, actions are monitored against the plan’s targets and most or all prescribed activities are completed | 12 |
| Boundary demarcation | METT | Scored 0 – 3:  0: The boundary of the protected area is not known by the management authority or local residents/neighbouring land users  1: The boundary of the protected area is known by the management authority but is not known by local residents/neighbouring land users  2: The boundary of the protected area is known by both the management authority and local residents but is not appropriately demarcated  3: The boundary of the protected area is known by the management authority and local residents and is appropriately demarcated | 12 |
| Law enforcement | METT | Scored 0 – 3:  0: The staff have no effective capacity/resources to enforce protected area legislation and regulations  1: There are major deficiencies in staff capacity/resources to enforce protected area legislation and regulations (e.g. lack of skills, no patrol budget)  2: The staff have acceptable capacity/resources to enforce protected area legislation and regulations but some deficiencies remain  3: The staff have excellent capacity/resources to enforce protected area legislation and regulations | 12 |
| Visitor Facilities | METT | Scored 0 – 3:  0: There are no visitor facilities and services  1: Visitor facilities and services are inappropriate for current levels of visitation or are under construction  2: Visitor facilities and services are adequate for current levels of visitation but could be improved  3: Visitor facilities and services are excellent for current levels of visitation | 12 |
| Research | METT | Scored 0 – 3:  0: There is no survey or research work taking place in the protected area  1: There is some *ad hoc* survey and research work  2: There is considerable survey and research work but it is not directed towards the needs of protected area management  3: There is a comprehensive, integrated programme of survey and research work, which is relevant to management needs | 12 |
| Monitoring and evaluation (M&E) | METT | Scored 0 – 3:  0: There is no monitoring and evaluation in the protected area  1: There is some *ad hoc* monitoring and evaluation, but no overall strategy and/or no regular collection of results  2: There is an agreed and implemented monitoring and evaluation system but results are not systematically used for management  3: A good monitoring and evaluation system exists, is well implemented and used in adaptive management | 12 |
| Condition assessment | METT | Scored 0 – 3:  0: Important biodiversity, ecological and cultural values are being severely degraded  1: Some biodiversity, ecological and cultural values are being severely degraded  2: Some biodiversity, ecological and cultural values are being partially degraded but the most important values have not been significantly impacted  3: Biodiversity, ecological and cultural values are predominantly intact | 12 |
